# Erratum

**DOI:** 10.1590/1518-8345.0000.3750

**Published:** 2022-08-01

Regarding the article “Impact of digital social media on the perception of loneliness and social isolation in older adults”, with DOI number: http://www.doi.org/10.1590/1518-8345.5641.3526, published in the Rev. Latino-Am. Enfermagem, 2022;30:e3526, page 1: 

Where was written: 

“Original Article” 

Now read: 

“Review Article” 

Regarding the article “Effect of thermometry on the prevention of diabetic foot ulcers: a systematic review with meta-analysis”, with DOI number: http://www.doi.org/10.1590/1518-8345.5663.3525, published in the Rev. Latino-Am. Enfermagem, 2022;30:e3525, page 1: 

Where was written: 

“Original Article” 

Now read: 

“Review Article”

